# Polarization of ADAM17‐driven EGFR signalling in electric field‐guided collective migration of epidermal sheets

**DOI:** 10.1111/jcmm.16019

**Published:** 2020-11-08

**Authors:** Naixin Jia, Jie Liu, Guoqin Zhu, Yi Liang, Yuan Wang, Weiyi Wang, Ying Chen, Jinrui Yang, Wangjun Zhang, Jiaping Zhang

**Affiliations:** ^1^ Key Laboratory of Freshwater Fish Reproduction and Development Ministry of Education Laboratory of Molecular Developmental Biology School of Life Sciences Southwest University Chongqing China; ^2^ Department of Plastic and Aesthetic Surgery State Key Laboratory of Trauma, Burns and Combined Injury Southwest Hospital The Third Military Medical University(Army Medical University) Chongqing China; ^3^ Dalian Rehabilitation Recuperation Center of PLA Joint Logistics Support Force Dalian China; ^4^ Department of Pancreatic and Biliary Surgery The First Affiliated Hospital of Harbin Medical University Heilongjiang China

**Keywords:** ADAM17, collective directional migration, EGFR, electric fields, F‐actin, HB‐EGF, wound healing

## Abstract

Endogenous electric field is considered to play an important role in promoting collective migration of epidermis to the wound centre. However, most studies are focused on the effect of bioelectric field on the movement and migration of single epithelial cell; the molecular mechanisms about collective migration of epidermal monolayers remain unclear. Here, we found that EFs dramatically promoted the collective migration of HaCaT cells towards the anode, activated the sheddase activity of ADAM17 and increased the phosphorylation level of EGFR. Moreover, EGFR phosphorylation and HB‐EGF shedding level were significantly decreased by the ADAM17 inhibitor TAPI‐2 or siADAM17 under EFs, which subsequently attenuated the directed migration of HaCaT sheets. Notably, the inhibition of EF‐regulated collective migration by siADAM17 was rescued by addition of recombinant HB‐EGF. Furthermore, we observed that F‐actin was dynamically polarized along the leading edge of the migrated sheets under EFs and that this polarization was regulated by ADAM17/HB‐EGF/EGFR signalling. In conclusion, our study indicated that ADAM17 contributed to the collective directional movement of the epidermal monolayer by driving HB‐EGF release and activating EGFR under EFs, and this pathway also mediated the polarization of F‐actin in migrating sheets, which is essential in directional migration.

## 1 INTRODUCTION

Re‐epithelialization of wounds involves several keratinocyte functions: proliferation, migration and differentiation. The key step is keratinocyte migration, an essential aspect for understanding chronic non‐healing wound.^1^ Directional migration of keratinocytes toward the wound centre is response to various physical and chemical factors in wound microenvironment. Among them, the endogenous electric fields are believed to play an important role in promoting the directional migration of epidermal cells to the wound centre.^2^ In wounds of epidermal tissue, endogenous EFs are naturally generated due to the collapse of transepidermal potentials (TEPs), causing the wounded centre to become more negative than the surrounding area, and the electric signals can override other cues to promote electrotactic responses, a phenomenon called cell electrotaxis/galvanotaxis.^3^

Numerous in vitro experiments confirmed the EF‐induced directional migration in many cell types such as corneal epithelial cells, endothelial cells, keratinocytes, and fibroblasts. A number of researchers investigated the biomolecular intracellular signalling pathways to reveal how the cells sense and control the polarity in response to the directional electric cue at a single‐cell level. The intracellular ‘compass model’ suggests a competition between the PI3K‐dependent pathway at the front and the myosin‐dependent pathway at the rear of the cell that determines the direction of single‐cell migration by the active formation of lamellipodia in directional response to the applied EF.^4^ EF was shown to induce a polarized activation of several other signalling pathways such as phosphatase and tensin homologue (PTEN), epidermal growth factor (EGF) receptors, mitogen‐activated protein kinase (MAPK), extracellular‐signal‐regulated kinase (ERK) and RhoA.^5^

The limitation of current knowledge is that the studies on the electrotactic response dealt with the cells that are in isolation. However, cellular motility in wound conditions concerns with the collective migration and it is unclear how an electric field will affect collective migration. Severe previous studies have demonstrated polarization of cell surface EGFR that coincides with the direction of electrotactic cell migration.^6^ In addition, EGFR has been implicated in regulating galvanotaxis in several cell populations, including fibroblasts, keratinocytes and other epithelial cells.^7^ In human keratinocytes, it has been reported that EGFR polarization occurred as early as 5 minutes after exposure to electric fields; inhibition of EGFR abolished not only the EGFR redistribution, but also the electrotactic migration.^8^ EF induced EGFR phosphorylation in a ligand‐independent manner, and subsequent inhibition of EGFR activity restrained galvanotaxis. Although EGFR is functional in collective migration, and more importantly, how it is activated by EFs has not been identified yet.

Recently, ADAM17 has been proved as the principal activators of the EGFR activation during wound repair. ADAM17 was identified as a major sheddase responsible for the ectodomain shedding of the multiple membrane‐bound EGFR ligand precursor, including heparin‐binding EGF‐like growth factor (HB‐EGF), transforming growth factor‐α (TGF‐α) and amphiregulin (AREG) that activate epidermal growth factor receptor (EGFR), which plays a significant role in wound healing.^9^ Thus, it is possible that EGF/EGFR pathway regulated by ADAM17 and thereby conducing directional migration of epithelial sheets.

The impact of ADAM17 on the directional collective migration of the epidermal monolayer by the application of physiological‐level EFs has not been assessed. We assume that directional collective migration of epidermal sheets under EFs is associated with increased activity of ADAM17 and thereby activation of HB‐EGF/EGFR signalling pathway. In the current study, we demonstrate that the HaCaT monolayer shows marked collective electrotactic migration and that its electrotactic response is regulated by ADAM17. This effect is dependent on activation of the HB‐EGF/EGFR signalling pathway. We also show that EF‐inducing F‐actin polarization is mediated by the ADAM17/HB‐EGF/EGFR signalling pathway, is consistent with directional migration of epidermal monolayer.

## 2 MATERIALS AND METHODS

### 2.1 Cell culture

The HaCaT cells were obtained from the Cell Bank of the Chinese Academy of Sciences in Beijing, China. Cells were cultured in RPMI 1640 medium supplemented with 100 μg/ml streptomycin, 100 U/ml penicillin and 10% foetal bovine serum (FBS). The HaCaT cells were incubated at 37°C in 95% humidity and 5% CO_2_.

### 2.2 EF stimulating and time‐lapse image recording

To observe HaCaT cells migration, the previously developed experimental electrotaxis chamber was utilized, as described.^10^, ^11^ To stimulate cells, EFs were fabricated through two silver electrodes immersed in Steinberg's solution‐filled reservoirs, which were connected to the culture medium by two agar bridges (2% agar in Steinberg's solution). Before EF stimulation, a CO2‐independent culture medium was transferred to the prepared chamber. Cell monolayer was exposed to EFs with electrical strengths ranging from 0 to 200 mV/mm for 6 h. Time‐lapse imaging was performed with a Zeiss imaging system (Carl Zeiss Meditec, Jena, Germany) to monitor migration of the cell monolayer, and images were recorded every 5 min for 6 h. Images analysis were implemented by using NIH ImageJ software.

### 2.3 Quantitative analysis of cell migration velocity and directedness

Directional cell migration was quantified using methods reported previously.^10^, ^12^ The directedness of cells was quantified by cos θ, which represents the angle between the field vector and the cell migration direction. Trajectory speed (Tt/t) was used to quantify the migration speed of cells, where (Tt/t) is the total length of the migration trajectory of a cell divided by the period of time. The displacement speed (Td/t) was the straight‐line distance between the start and end positions of a cell divided by the period of time, and the x‐axis was calculated as the cell's displacement distance along the EF vector divided by the period of time.

### 2.4 ADAM17 activity assay

ADAM17 activity was determined by an InnoZyme TACE/ADAM17 (α‐Secretase) Activity Kit (Calbiochem) according to the manufacturer's protocol. Briefly, each group of cell extracts was first incubated in anti‐human ADAM17 coated 96‐well plates for 1 hour and then incubated with ADAM17 fluorescently labelled substrate for 4‐5 hours. The relative fluorescence of internally cleaved products was measured at an excitation wavelength of ~324 nm and emission wavelength of ~410 nm. All data were normalized with respect to data from the control group.

### 2.5 Transfection of ADAM17 siRNA and pharmacological reagents

ADAM17‐small interference RNA (target sequence of 5′‐CCAUGAACACGUGU‐3′) and a negative control (NC) siRNA (target sequence of 5′‐UUACACGUGUUCUUCAUGGT‐3′) were purchased from GenePharma (China). In brief, the cell monolayer was incubated in penicillin‐ and streptomycin‐free culture medium involved in 0.1 μM ADAM17‐siRNA or NC siRNA for 24 hours. Western blot and immunofluorescence were used for evaluating ADAM17 expression. Cell monolayer was treated with ADAM17 inhibitor TAPI‐2 (Sigma CAS 187034‐31‐7, USA) at a final concentration of 40 μM, EGFR inhibitor AG1478 (Calbiochem, La Jolla, CA) at a final concentration of 10 μM and recombinant human heparin‐binding EGF (R&D Systems, Wiesbaden‐Nordenstadt, Germany) at a final concentration of 100 ng/ml.

### 2.6 Western blot analysis

The cell monolayer was washed with ice‐cold phosphate‐buffered saline (PBS), harvested in 50‐200 μL lysis buffer. The lysates were sonicated for 4 seconds and separated by centrifugation at 14 000 ***g*** for 15 min at 4°C. The protein concentration was determined using a bicinchoninic acid (BCA) protein assay kit (Sigma, USA). Lysates containing equal amounts of proteins were separated on a 10% SDS‐PAGE and transferred electrophoretically onto polyvinylidene difluoride (PVDF) membranes. The membranes were blocked in 5% bovine serum albumin (BSA) (Sigma‐Aldrich) in TBST for 3 hours at room temperature. The blots were probed with primary antibodies at 4°C overnight. The above primary antibodies were used at a 1:1000 dilution, the loading control anti‐β‐actin at a 1:4000 dilution and the secondary antibody at a 1:4000 dilution. The membranes were probed using the following primary antibodies: anti‐β‐actin (Proteintech, USA), anti‐ADAM17 (Abcam ab39162, UK), anti‐EGFR (Abcam ab52894, UK) and anti‐p‐EGFR (Abcam ab40815, UK). The images were quantified with the Quantity One 4.1 software (Bio‐Rad, USA).

### 2.7 ELISA

To evaluate the release of ADAM17 substrates, the cell monolayer was stimulated by an EF for 6 h, and then, the supernatant was collected. Relevant DuoSet ELISA kits (Cloud‐clone Corp, HB‐EGF SEB479Hu, AREG SEA006Hu, TGF‐α SEA123Hu, China) were adopted to analyse the supernatant.

### 2.8 Immunofluorescence

Following EF‐directed HaCaT cells migration, the cells were fixed with 4% paraformaldehyde for 20 minutes at room temperature. After washing with PBS three times, the cell monolayer was blocked with 5% BSA (Sigma‐Aldrich). Staining F‐actin with rhodamine phalloidin (Proteintech, USA) at a 1:50 dilution for 2 hours. After washing with PBS, the cell monolayer was mounted with DAPI (Sigma F6057, USA). Images were obtained using a Leica confocal microscope (Leica Microsystems, Wetzlar, Germany).

### 2.9 Statistical analysis

The values are expressed as the mean ± SEM. The results were compared by analysis of variance for repeated measurements, followed by Student‐Newman‐Keuls t test. Comparisons in different groups were performed by using Tukey's honestly significant difference (HSD) test or Dunnett's test. *P < *.05 was considered statistically significant.

## 3 RESULTS

### 3.1 Collective electrotaxis of epithelial monolayers under EF

**Figure 1:**
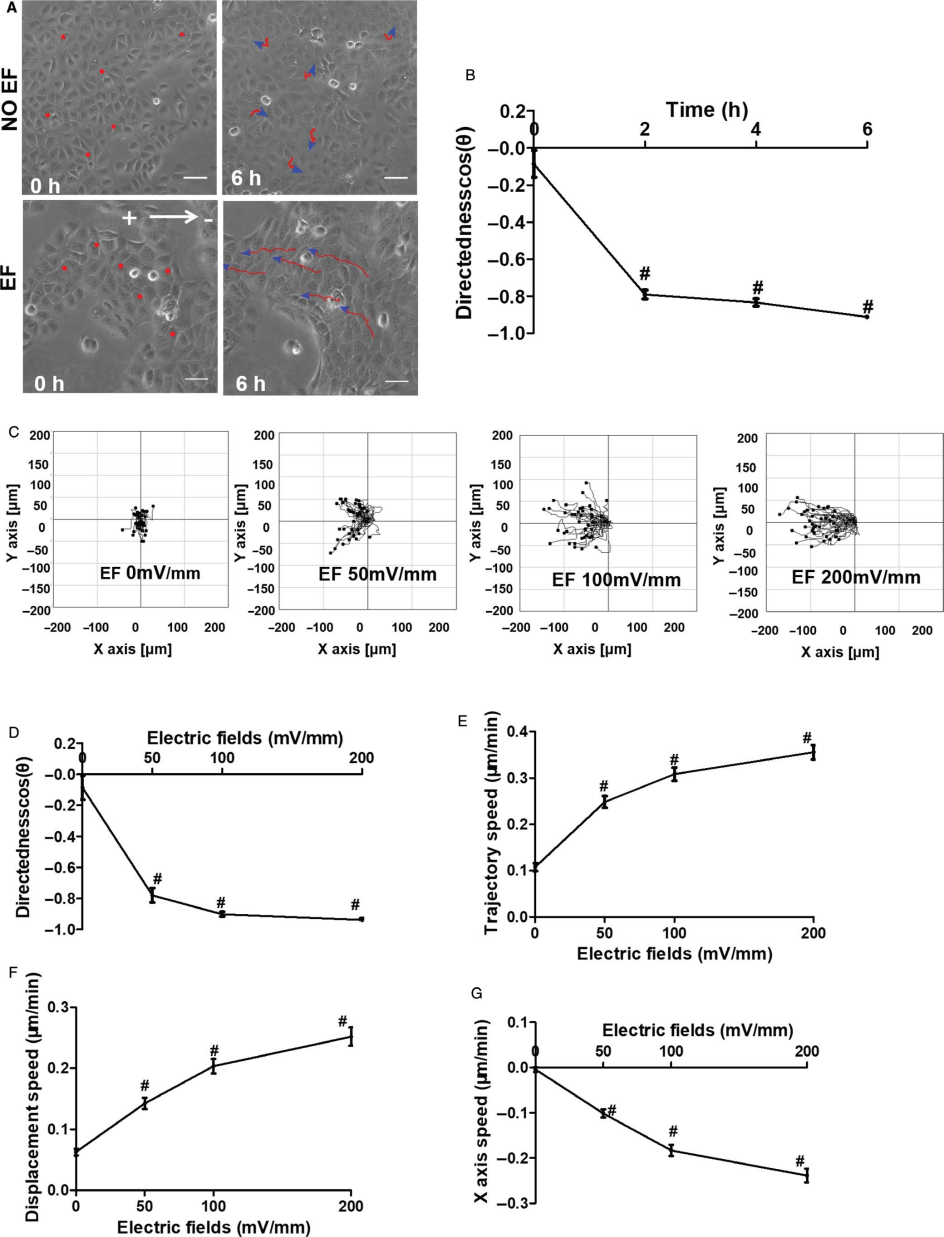
Electric fields stimulated directional collective migration of HaCaT monolayer. A, Time‐lapse images showing the electrotactic response of the HaCaT monolayer; (B) migratory directedness of HaCaT monolayer in EFs of different times; (C) migration trajectories of HaCaT monolayer under EFs; (D) migratory directedness of HaCaT monolayer in EFs of different voltages; (E‐G) trajectory speed, displacement speed and x‐axis speed of HaCaT monolayer in EFs of different voltages. Arrow indicates the direction of the electric field. Data were obtained from at least three independent experiments and are shown as the mean ± SEM. #*P* < .05 vs. the no EF group. Scale bars 50 μm

To evaluate the electrotactic behaviour of the epidermal monolayer, we first observed EF‐stimulated migration of the HaCaT cells by time‐lapse microscopy in the absence or presence of an EF. In the absence of an EF, no obvious directional migration was observed. The cells in a confluent monolayer migrated very little and randomly. In an EF of 100 mV/mm, the cells migrated towards the anode collectively, at an increased speed (Figure 1A, Movie S1). Although the cell monolayer migrated collectively and directionally, the single cell maintained their relative position in a monolayer, and the leading cells were seen to extend lamellipodia and filopodia towards the anode (Movie S1). This directional response, as indicated by the value of directedness, could be detected at 30 minutes, peaking at 120 minutes following the onset of the EF, indicating a time‐dependent manner for EF‐induced collective migration of epidermal monolayer (Figure 1B).

We next determined whether the collective directional response was dependent on the EF strength. In the presence of EFs ranging from 50 to 200 mV/mm, significant directional collective migration of cells could be observed with directedness of −0.78 ± 0.031, −0.90 ± 0.014 and −0.94 ± 0.009, respectively. (Figure 1C‐D) (*P* < .01, when compared with No EF control). The threshold voltage required to induce electrotaxis of cell monolayers was therefore largely lower than 50 mV/mm. Applied EFs also significantly increased the trajectory speed. The increase in speed was also dependent on field strength, with an EF of 200 mV/mm increasing the speed by 231% (Figure 1E). Consistent with the directional migration, displacement along the χ‐axis (the field line towards the anode) in cells also significantly increased following EFs stimulation (Figure 1F‐G). These results suggest a robust electrotactic response in epithelial monolayer in a time‐ and voltage‐dependent manner.

### 3.2 ADAM17 is required for EF‐induced collective directional migration of the epidermal monolayer

**Figure 2:**
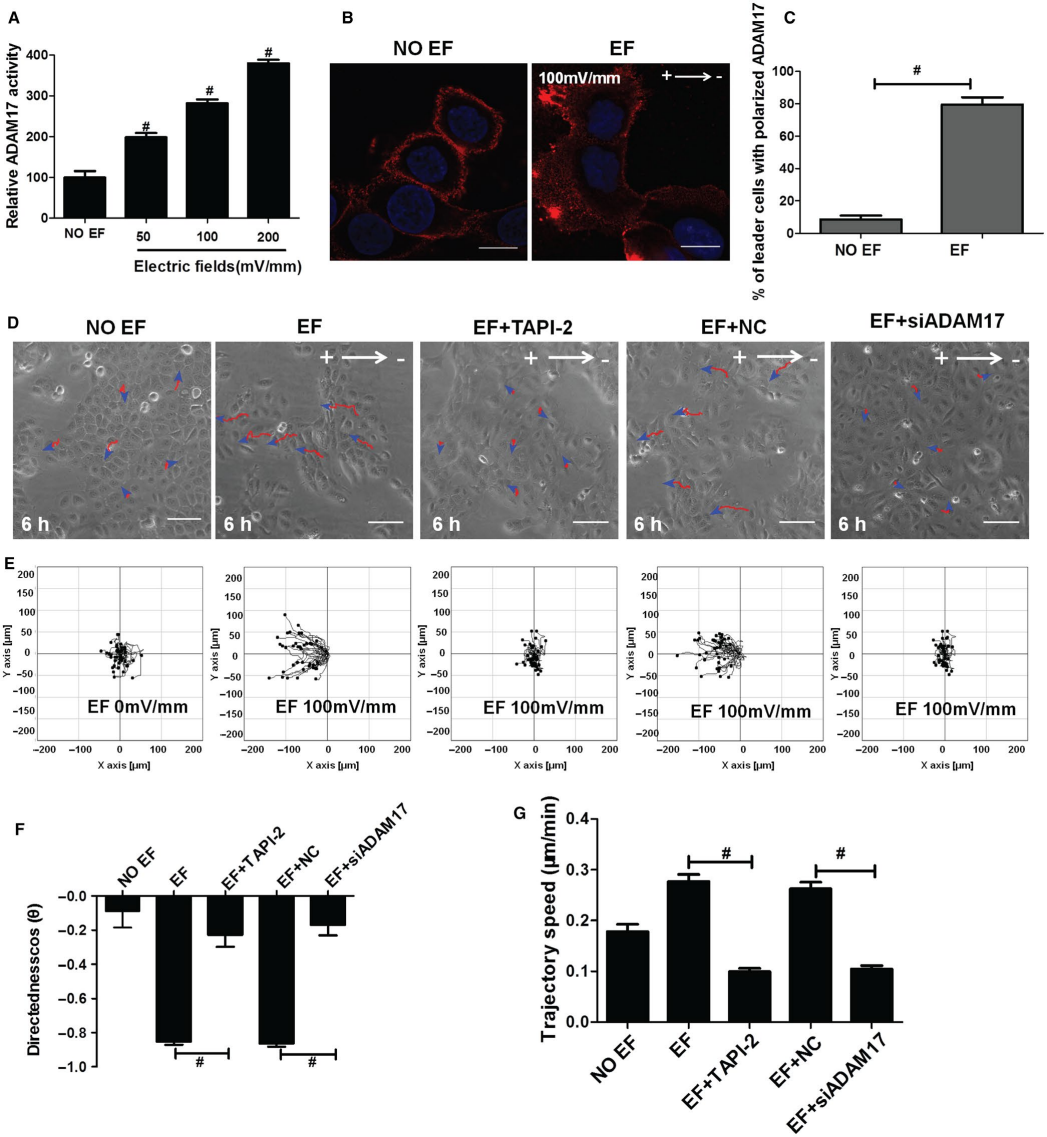
Down‐regulation of ADAM17 significantly impaired EF‐induced collective directional migration of the epidermal monolayer. A, A TACE activity assay kit was used to measure ADAM17 activity of HaCaT monolayer under EFs. B, Fluorescence confocal images showing the staining and distribution of ADAM17 in the absence of an EF or in the presence of an EF. C, Statistical analysis indicating the ADAM17 distribution in the absence of an EF or in the presence of an EF. D, Representative images of HaCaT monolayer at 6 h after EF stimulation in various groups. E, Migration trajectories of HaCaT monolayer at 6 h after EF stimulation in various groups. F‐G, Effect of the ADAM17 inhibitor TAPI‐2 or si‐ADAM17 on migration directedness and trajectory speed. Arrow indicates the direction of the electric field. Composite images (B) are merged images consisting of 2 channels: ADAM17 (red) and DAPI (blue). Data were obtained from at least three independent experiments and are shown as the mean ± SEM. #*P* < .05 vs. the EF group. *Scale bars* (B) 10 µm, (D) 50 μm

To determine whether ADAM17 contributes to the collective directional migration of epidermal monolayers under EFs, we first examined the enzymatic activity and distribution of ADAM17 in a HaCaT monolayer after EFs application. As shown in Figure 2A, ADAM17 activity was markedly increased by EFs. In an EF of 50 mV/mm, ADAM17 activity of cell monolayer was increased (0 mV/mm: 99.3504 50 mV/mm: 198.013) by 99% compared to No EF control. EFs of higher voltage further increased ADAM17 activity, which was about (200 mV/mm: 379.973) 2.8‐fold of the control in an EF of 200 mV/mm. Interestingly, by immunofluorescence staining we found that ADAM17 showed an asymmetrical redistribution in epidermal monolayer under EF stimulation (100 mV/mm) (Figure 2B). Although ADAM17 distributed at cell membrane equably in No EF‐treated monolayer, it redistributed mainly at the extended lamellipodia and filopodia in the leading cells that were located at the anodal side of cell monolayer after EF application. Compared with No EF group, the ADAM17 polarization towards the anode increased significantly from 9% to 80% under EFs at the leading edge (Figure 2B,C). These results suggest that EFs have significant effects on ADAM17.

Next, we examined a possible role of ADAM 17 in EF‐induced collective directional migration of HaCaT cell monolayer by pretreatment of TAPI‐2, a specific ADAM17 inhibitor or siADAM17, a specific ADAM17‐small interference RNA. Immunofluorescence staining and immunoblot analysis all confirmed a complete depletion of ADAM17 by siADAM17 transfection (Figure S2). Strikingly, we found that both TAPI‐2 and si‐ADAM17 caused profound reductions in EF‐induced directional collective migration of cells (Figure 2D,E; Movie S3‐S4). The migration directedness in TAPI‐2 and si‐ADAM17 pretreated monolayers declined by 74% and 80%, respectively, when compared to controls (Figure 2F). Trajectory speed also significantly decreased by TAPI‐2 and si‐ADAM17 pretreatments (Figure 2G). Morphologically, no extended lamellipodia and filopodia in the leading cells at the anodal side of cell monolayer were observed by TAPI‐2 and si‐ADAM17 pretreatment (Movie S3‐S4). Furthermore, cells seemed to loss close cell‐cell contacts in si‐ADAM17, but not TAPI‐2 pretreated monolayer, indicating an implication of ADAM17 in cell‐cell adhesions independent of its sheddase activity (Figure 2D). Nevertheless, our data provided clear evidence showing an essential role for ADAM17 activation in EF‐induced collective directional migration of epidermal sheets.

### 3.3 ADAM17 contributes to EF‐directed collective migration through EGFR signalling

**Figure 3:**
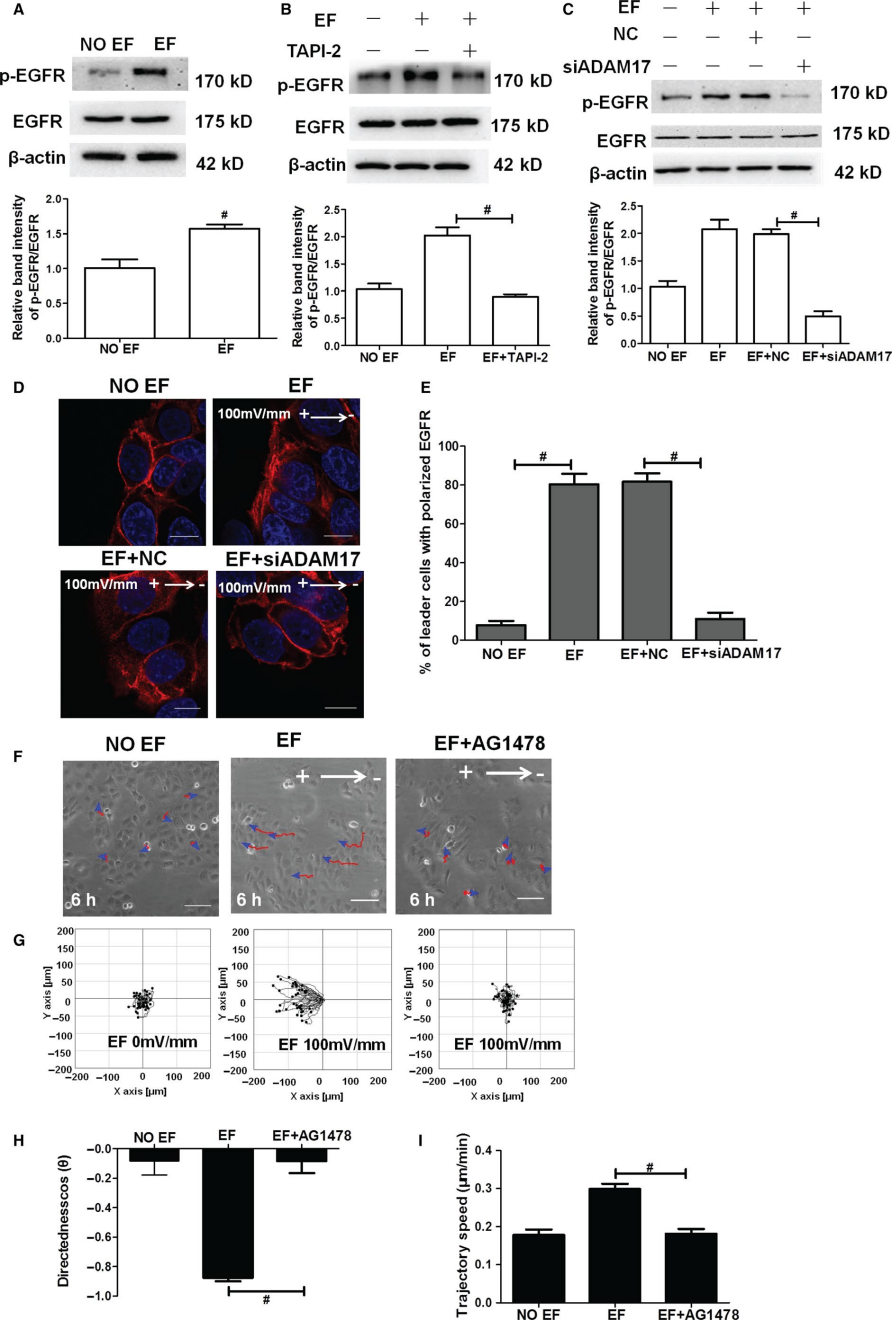
ADAM17‐induced EGFR activation promotes the collective directional migration of the epidermal monolayer under EFs. A, Representative immunoblotting and quantification of p‐EGFR in HaCaT monolayer exposed to EFs. B, Representative immunoblotting and quantification of p‐EGFR in HaCaT monolayer exposed to TAPI‐2 under EFs. C, Representative immunoblotting and quantification of p‐EGFR in HaCaT monolayer exposed to si‐ADAM17 under EFs. D, Fluorescence confocal images showing the staining and distribution of EGFR after EF exposure without drugs or with siRNA‐ADAM17 treatment. E, Statistical analysis indicating the EGFR distribution after EF exposure without drugs or with siRNA‐ADAM17 treatment. F, Representative images of HaCaT monolayer at 6 h after EF stimulation in various groups. G, Migration trajectories of HaCaT monolayer at 6 h after EF stimulation in various groups. H‐I, Effect of the EGFR inhibitor AG1478 on migration directedness and trajectory speed. Arrow indicates the direction of the electric field. Composite images (D) are merged images consisting of 2 channels: EGFR (red) and DAPI (blue). Data were obtained from at least three independent experiments and are shown as the mean ± SEM. #*P* < .05 vs. the EF group. Scale bars (D) 10 µm, (F) 50 μm

As ADAM17 has been proven as a major sheddase responsible for the ectodomain shedding of the multiple membrane‐bound EGFR ligand precursor, and EGFR has been identified to control the electrotactic response of epithelial cells, we then hypothesized that ADAM17 may contribute to the EF‐induced collective directional migration of the cells via EGFR. To test our hypothesis, we firstly examined the status of EGFR activation (tyrosine‐phosphorylated EGFR) during ADAM17‐regulated electrotactic responses of the epidermal monolayer. As shown in Figure 3A, EGFR phosphorylation was strongly increased in EF‐treated monolayer as compared to No EF control. The EF‐induced EGFR phosphorylation could be totally abolished by TAPI‐2 or siADAM17 pretreatment, indicating a crucial role for ADAM17 in EF‐induced EGFR activation (Figure 3B,C). Morphologically, we also found that EGFR showed a strong staining in the extended lamellipodia and filopodia at the anodal side of the leading cells after exposure to the electric field (Figure 3D), similar to the ADAM 17 staining under EFs (Figure 2B). This polarization staining of EGFR could be totally disrupted by siADAM17 pretreated, indicating that not only the activation, but also the asymmetrical redistribution of EGFR under EFs was controlled by ADAM17 (Figure 3D,E). Accordingly, we observed an obvious reduction in the collective electrotactic response in HaCaT monolayer exposed to AG1478, an EGFR tyrosine kinase inhibitor (Figure 3F,G; Movie S5). With exposure to EGFR inhibitor, the mean directedness of cells decreased from −0.88 to −0.08 (Figure 3H), and the mean trajectory speed decreased from 0.30 μm/min to 0.18 μm/min (Figure 3I). Inhibition of EGFR virtually abolished the electrotactic response of epithelial sheets. These data suggest that ADAM17 facilitates the EF‐induced collective directional migration of cells through EGFR signalling.

### 3.4 HB‐EGF plays a key role in ADAM17/EGFR axis‐mediated collective directional migration under EFs

**Figure 4:**
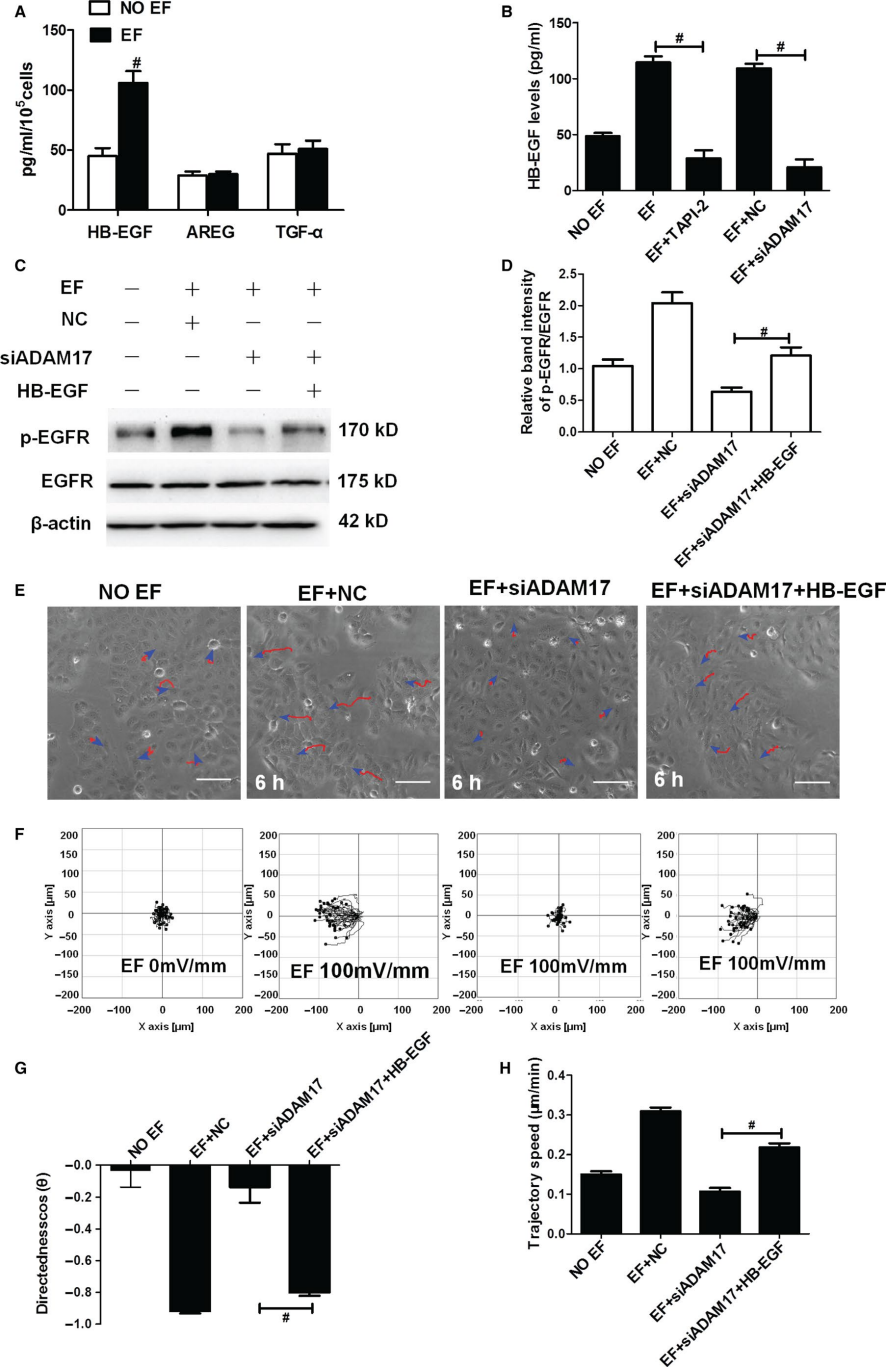
HB‐EGF plays a significant role in the collective directional migration of the epidermal monolayer regulated by the ADAM17/EGFR pathway under EFs. A, The release of EGF molecules, including HB‐EGF, AREG and TGF‐α, in the epidermal monolayer by application of an EF. B, The release of HB‐EGF was strongly reduced by siADAM17 in HaCaT monolayers under EFs. C‐D, Representative immunoblotting and quantification showing the effect of recombinant HB‐EGF on p‐EGFR in ADAM17‐silenced epidermal monolayer treated with EFs. E, Representative images taken from HaCaT monolayer after EF stimulation in various groups. F, Migration trajectories of HaCaT monolayer after EF stimulation in various groups. G‐H, Effect of recombinant HB‐EGF on the migration directedness and trajectory speed of the epidermal monolayer. Arrow indicates the direction of the electric field. Data were obtained from at least three independent experiments and are shown as the mean ± SEM. #*P* < .05 vs. the EF + siADAM17 group. Scale bars 50 μm

In ADAM17 active epidermis, EGF family molecules such as HB‐EGF, TGF‐α and amphiregulin (AREG) are released, which contribute to the re‐epidermalization and accelerates wound healing. To identify which EGF molecules play a key role in the ADAM17/EGFR axis‐mediated collective directional migration under EFs, we examined the release of EGF molecules in HaCaT cell monolayer exposed to EFs. The ELISA results showed that only the HB‐EGF levels in culture supernatants were markedly increased (Figure 4A). Pretreatment with TAPI‐2 or siADAM17 totally abolished the elevated release of HB‐EGF from electrotactic cells (Figure 4B), confirming a role of ADAM17 in HB‐EGF shedding. Although the EF‐induced EGFR phosphorylation was prohibited by siADAM17, it could be partially restored by the adding of recombinant HB‐EGF (Figure 4C‐D). To further elucidate whether HB‐EGF plays a crucial role in the ADAM17/EGFR axis‐mediated electrotactic response, we detected the effects of the recombinant HB‐EGF in ADAM17‐silenced HaCaT cells. As expected, the EF‐induced electrotactic migration of HaCaT cell monolayer was prohibited by si‐ADAM17. This prohibited electrotactic migration, however, was largely rescued by the subsequent addition of recombinant HB‐EGF (Figure 4E,F; Movie S6): the directedness increased from −0.13 to −0.80 (Figure 4G), and the trajectory speed restored partially (Figure 4H). These results, taken together, suggest that HB‐EGF plays a pivotal role in ADAM17/EGFR axis‐mediated collective electrotactic migration in epidermal monolayer.

### 3.5 F‐actin polarization is controlled by the ADAM17/EGFR axis in epidermal monolayer exposed to EFs

**Figure 5:**
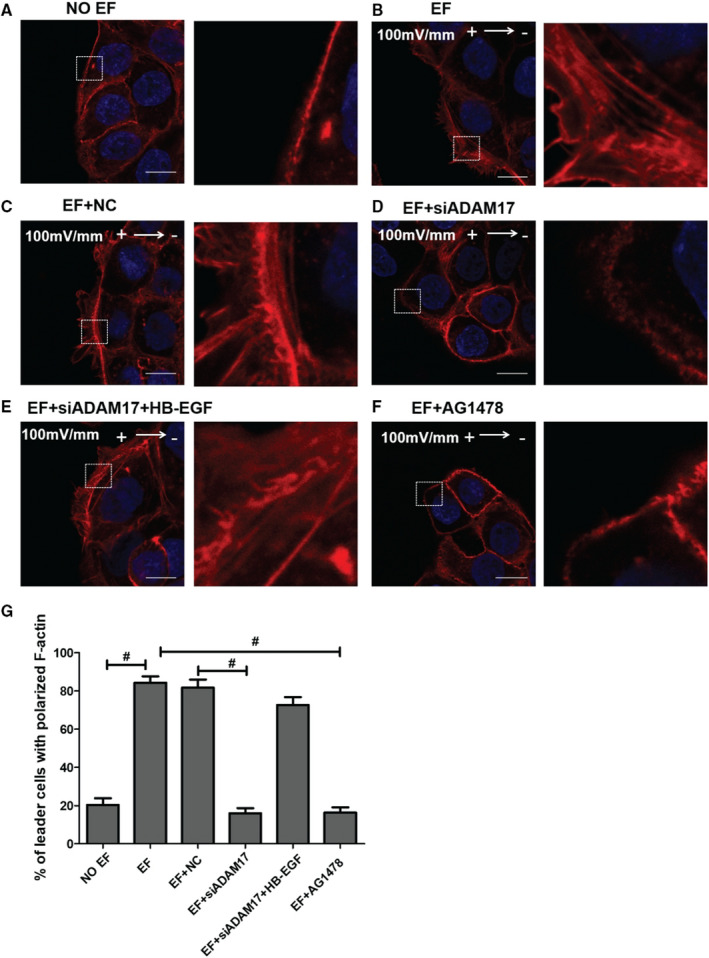
Effect of the ADAM17/HB‐EGF/EGFR signalling pathway on EF‐induced F‐actin polarization in the epidermal monolayer. A‐B, Fluorescence confocal images showing the staining and distribution of F‐actin in the absence of an EF (A) or in the presence of an EF (B); (C‐D) effects of NC and siADAM17 on the F‐actin asymmetric rearrangement of HaCaT cells treated by an EF; (E) effects of recombinant HB‐EGF on F‐actin polarization of ADAM17‐silenced cells under application of an EF; (F) effect of AG1478 on the F‐actin asymmetric redistribution of cells under an EF. (G) Statistical analysis indicating the F‐actin distribution of cells in various groups. Arrow indicates the direction of the electric field. Composite images (A‐F) are merged images consisting of 2 channels: F‐actin (red) and DAPI (blue). Data were obtained from at least three independent experiments and are shown as the mean ± SEM. #*P* < .05 vs. the EF group. Scale bars 10 μm

Cell migration is mediated by the protrusion of cytoplasm termed lamellipodia and filopodia. Protrusion is driven by actin polymerization, a process that is regulated by signalling complexes. The importance of F‐actin polarization at the leading edge of migrating sheets in the initiation and maintenance of the EF‐directed collective migration has been confirmed in epidermal cells.^13^ Here, we investigated whether the EF‐directed F‐actin polarization is regulated by the ADAM17/EGFR axis. As compared to No EF control, F‐actin was found to localize to the extended lamellipodia and filopodia in the leading cells at anodal side of cell monolayer in an EF, confirming F‐actin polarization induced by EFs, the F‐actin polarization towards the anode increased significantly from 20% to 84% (Figure 5A, B and G). siADAM17 pretreatment disrupted the EF‐induced protrusions as well as the asymmetric rearrangement of F‐actin, the F‐actin polarization towards the anode declined by 66% when compared to EF NC group ( Figure 5C, D and G). This disruption, however, could be dynamically reproduced by the addition of recombinant HB‐EGF (Figure 5E). Pretreatment with AG1478, a specific EGFR inhibitor, also resulted in disappearances of F‐actin polymerization and cell protrusions at the leading edge of the cell monolayer, the F‐actin polarization towards the anode declined by 68% when compared to EF group (Figure 5B, F and G). Taken together, these results suggest a crucial role for ADAM17 in F‐actin polarization through controlling the shedding of HB‐EGF and the subsequent activation of EGFR signalling at the leading cells in an electrotactic monolayer.

## 4 DISCUSSION

**Figure 6:**
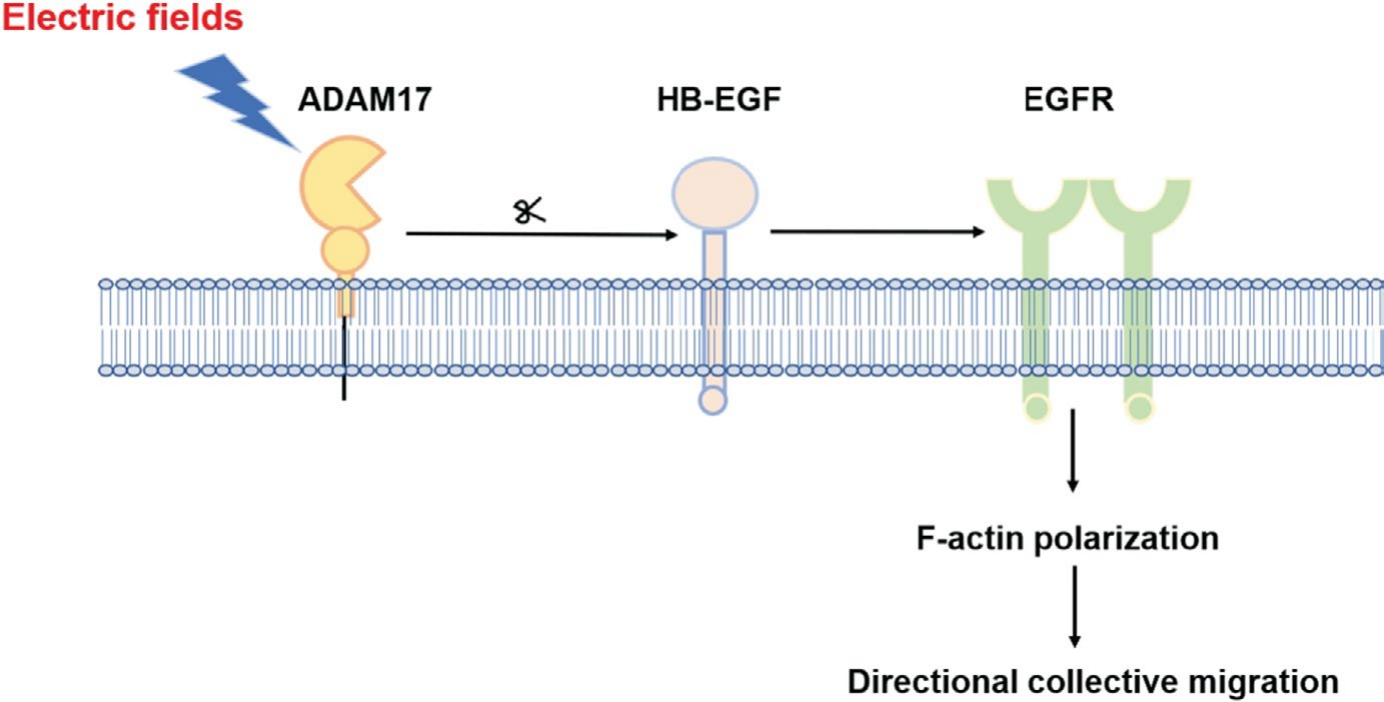
Schematic model depicting the role of ADAM17/HB‐EGF/EGFR signalling pathway in directional collective migration of epidermal cells

In this study, we demonstrated that EF promotes the directional migration of epidermal sheets through the activation and polarization of ADAM17/EGFR axis. In this axis, HB‐EGF, controlled by ADAM17 shedding, leads to EGFR activation and the subsequent F‐actin polarization, gearing up a directional collective migration of epidermal monolayer (Figure 6).

In vivo, epidermal cells migrate to wounds collectively in a uniform manner. Many candidate cues have been suggested directing cell migration during wound healing, of which the endogenous EFs have been proposed as a particularly important one.^14^ Endogenous EFs are generated instantaneously after an injury due to the collapse of the trans‐epithelial potentials. In vitro, many cell types respond to applied EFs at the strength equivalent to those measured in vivo (40‐200 mV/mm) by directional migration, a phenomenon termed electrotaxis or galvanotaxis.^15^ Significantly, recent studies have found that EFs could override other cues in guiding cell migration during epidermal wound healing, indicating that the role for EFs in wound healing is far more important than previously thought.^16^

Although numerous studies have been conducted on EF‐guided migration of cells in isolation, it could not completely replicate wound healing in vivo where healing occurs through epidermal collective migration. Therefore, understanding the behaviour of epidermal monolayer under EFs would be of great clinic significance. In fact, recent studies have described the EF‐induced collective migration in cell sheets of keratinocyte, corneal epithelial and mammary epithelial cells.^17^, ^18^, ^19^ Compared with cells in isolation, the epithelial sheet exists some unique electrotactic characteristics. Particularly, epithelial sheet exhibits greater sensitivity, more effective electrotaxis and directional persistence responding to EFs.^17^, ^18^ In our study, we did find that EF at strength as low as 50 mV/mm induced a robust electrotactic response in epidermal monolayer with directedness of −0.78 ± 0.031 (Figure 1C). The electrotactic response was presented in a time‐ and voltage‐dependent manner (Figure 1). When compared to the isolated cells, the HaCaT sheets exhibited better electrotaxis with directedness 10.44‐fold and trajectory speed 2.86‐fold of the control under the same EF (Figure S1; Movie S2). These observations indicate some inherent discrepancies between isolated cell migration and cell collective migration.

The molecular mechanisms controlling directional collective migration remain largely unclear. Previous studies on isolated cells have suggested that the electrotactic response is regulated in a synergetic manner by signalling pathways such as EGFR/MAPK, PI3K/Akt and integrin signalling pathways.^13^, ^20^, ^21^, ^22^ In our study, we showed that EGFR is also essential for EF‐directed collective migration of HaCaT sheets. After exposure to EFs, EGFR was activated and concentrated at the site of lamellipodia and filopodia protrusions in leading cells; inhibition of EGFR totally abolished the electrotactic response. These observations suggest that the EGFR signalling might be a common pathway for EF‐induced electrotactic migration in either isolated cells or cell sheets. We for the first time identified that the activation and polarization of EGFR are tightly controlled by ADAM17, a main sheddase of EGFR ligands that was also asymmetrically redistributed and activated after EF stimulation. Particularly, ADAM17 inhibition or knockdown caused a similar reduction in electrotactic migration of cell sheets. These novel findings, therefore, declared a polarization of ADAM17‐driven EGFR signalling crucial for EF‐guided collective migration of epidermal sheets. Our finding could explain the observations in previous study where the electrotactic response is lost or attenuated in the absence of EGF in various cells, because ADAM17 is known as the major sheddase responsible for the ectodomain shedding of EGFR ligand HB‐EGF, TGF‐α and AREG.

The importance of ADAM17/EGFR axis in skin homeostasis has been established. ADAM17/EGFR signalling contributes to epidermal migration and re‐epithelialization during wound healing and also plays important roles in differentiation, skin barrier integrity and hair follicle bulge niche.^23^, ^24^ The mechanisms for the functional diversities of this axis remain unclear, but might be close related to the substrate specificity of ADAM17‐dependent shedding under different conditions. For example, Maretzky et al reported that iRhom2, a factor essential for ADAM17 maturation, is required for the stimulated shedding of HB‐EGF and Kit ligand 2, but not TGF‐α in mouse embryonic fibroblasts.^25^ In human HaCaT keratinocytes, it has been shown that melittin, the major component of the bee venom, induced shedding of E‐cadherin and TGF‐α followed by transactivation of EGF receptor and ERK1/2 phosphorylation.^26^ In our study, HaCaT cell monolayer exposed to EFs resulted in a marked increase in HB‐EGF, but not TGF‐α and AREG. By inhibition or silencing experiments, we confirmed a specific role for ADAM17 in HB‐EGF shedding. Adding of the recombinant HB‐EGF in ADAM17‐silenced cells restored the EF‐induced EGFR phosphorylation and rescued the directional collective migration. These results suggest a substrate specificity of ADAM17 shedding upon EFs stimulation, and the HB‐EGF is pivotal in ADAM17/EGFR axis‐mediated epidermal collective migration under EFs. However, the mechanism of ADAM17 activation by electric field remains unclear. Previous research reported endogenous electric fields (EFs) arise because of spatial and temporal variations in epithelial transport of charged ions such as Na+, K+ and Cl–, and spatial variations in the electrical resistance of epithelial sheets.^27^ The transport and rearrangement of these ions will inevitably have an important effect on the conformational changes of other charged groups on the cell membrane. Reiss et al reported that cationic amino acid residues in the membrane proximal domain (MPD) then interact with the negatively charged phosphatidylserine (PS)‐head group, bringing the protease into position for substrate processing, and trigger ADAM17‐mediated shedding of its substrates from the cell surface.^28^ The above researches provide a new enlightenment for us to further explore the mechanism of ADAM17 activation by electric field.

Effective directionality requires cooperation of numerous cellular movements such as the formation of lamellipodia and filopodia at the leading edge.^29^ The formation of lamellipodia and filopodia is driven by actin polymerization, a process regulated by signalling complexes. Previous work has shown that ADAM17 up‐regulation enhances actin cytoskeletal remodelling at the tip of the lamellipodium in HCC cells.^30^ Studies from Zhao and Fang et al have indicated a crucial role for HB‐EGF/EGFR signalling in F‐actin colocalization and polarization in a physiological EF.^8^, ^13^ In our study, we observed the polarization of ADAM17, EGFR and F‐actin in the electrotactic collective migration of cell sheets (Figures 2B, 3D and 5B). EGFR inhibition or ADAM17 silencing prohibited cell protrusions and F‐actin redistribution, along with the reduction of the electrotactic collective migration (Figure 5). These data provided cytoskeletal evidence further confirming the involvement of ADAM17/EGFR signalling axis in EF‐guided collective migration.

In conclusion, our findings indicate that EFs guide a robust directional collective migration in epidermal sheets. Moreover, ADAM17 is responsible for this obvious EF‐induced collective directional migration. Importantly, the above electrotactic migration mechanism is regulated by the HB‐EGF/EGFR signalling pathway. F‐actin is significantly redistributed towards the leading edge of migrating sheets, and F‐actin polarization is linked to the ADAM17/HB‐EGF/EGFR signalling pathway. Our study reveals novel findings related to the signalling mechanism of EF‐guided collective migration of epithelial cells, which could lead to new wound management strategies.

## CONFLICT OF INTEREST

The authors declare no competing financial or non‐financial interests.

## AUTHOR CONTRIBUTIONS

**Naixin Jia:** Data curation (equal); Formal analysis (lead); Investigation (equal); Methodology (lead); Project administration (equal); Software (equal); Validation (lead); Visualization (equal); Writing‐original draft (equal). **Jie Liu:** Data curation (equal); Formal analysis (equal); Methodology (equal); Software (equal); Writing‐original draft (equal). **Guoqin Zhu:** Formal analysis (supporting); Investigation (supporting); Methodology (supporting). **Yi Liang:** Formal analysis (supporting); Investigation (supporting); Methodology (supporting). **Yuan Wang:** Formal analysis (supporting); Investigation (supporting); Methodology (supporting). **Weiyi Wang:** Formal analysis (supporting); Methodology (supporting); Software (supporting). **Ying Chen:** Formal analysis (supporting); Methodology (supporting); Software (supporting). **Jinrui Yang:** Formal analysis (supporting); Methodology (supporting); Software (supporting). **Jun Wang Zhang:** Formal analysis (equal); Methodology (equal); Resources (equal); Software (equal). **Jiaping Zhang:** Conceptualization (lead); Data curation (lead); Formal analysis (equal); Funding acquisition (lead); Investigation (equal); Methodology (equal); Project administration (lead); Resources (lead); Software (equal); Supervision (lead); Validation (equal); Writing‐review & editing (equal).

## Supporting information

Figures S1‐S3
Movies S1‐S6

Video S1

Video S2

Video S3

Video S4

Video S5

Video S6

Fig S1

Fig S2

Fig S3
