# Supplementary material for: Polarization of ADAM17‐driven EGFR signalling in electric field‐guided collective migration of epidermal sheets
Source: J Cell Mol Med. 2020 Nov 8;24(23):14073–85. doi: 10.1111/jcmm.16019 (PMC7753989; doi:10.1111/jcmm.16019)
Supplement: Supplementary file 1 — Figures S1‐S3 Movies S1‐S6 [file JCMM-24-14073-s001.docx]

**Supporting Information**

**Fig. S1 HaCaT monolayer displayed higher migration efficiency than cells in isolation.** (A) Time-lapse images showing the electrotactic response of the HaCaT monolayer and isolated cells; (B) Migration trajectories of HaCaT monolayer and isolated cells under EFs; (C) Migratory directedness of HaCaT monolayer and isolated cells in EFs; (D) Trajectory speed of HaCaT monolayer and isolated cells in EFs. Arrow indicates the direction of the electric field. Data were obtained from at least three independent experiments and are shown as the mean ± SEM. #*p*<0.05 vs. the isolated cells. *Scale bars* 50 μm.

**Fig. S2** **ADAM17 expression in HaCaT cells after transfection with ADAM17-siRNA.** (A-B) Representative immunoblotting and quantification showing ADAM17 in HaCaT cells exposed to siADAM17. (C) Fluorescence confocal images showing the staining and distribution of ADAM17 in HaCaT cells exposed to siADAM17. Composite images (**C**) are merged images consisting of 2 channels: ADAM17 (**red**) and DAPI (**blue**). Data were obtained from at least three independent experiments and are shown as the mean ± SEM. #*p*<0.05 vs. the control group. *Scale bars* 10 μm.

**Fig.S3 The influence of ADAM17 in basal migration and electrotactic migration of epidermal monolayer.** (A) Migratory directedness of HaCaT monolayer with or without EF stimulation; (B) Trajectory speed of HaCaT monolayer with or without EF stimulation. Data were obtained from at least three independent experiments and are shown as the mean ± SEM. #*p*<0.05 vs. control group

**Supporting movie captions:**

**Supplemental movie 1**: Electric fields stimulated directional collective migration of HaCaT monolayer.

**Supplemental movie 2**: HaCaT cell monolayer displayed higher migration efficiency than cells in isolation.

**Supplemental movie 3**: ADAM17-inhibitor TAPI-2 inhibited collective directional migration of HaCaT cell monolayer.

**Supplemental movie 4**: siRNA-ADAM17 inhibited collective directional migration of HaCaT cell monolayer.

**Supplemental movie 5**: AG1478 (EGFR specific inhibitor) inhibited collective directional migration of HaCaT cell monolayer.

**Supplemental movie 6**: Recombinant HB-EGF promote the collective directional migration of the HaCaT cell monolayer.
