# Supplementary figures and images for: Polarization of ADAM17‐driven EGFR signalling in electric field‐guided collective migration of epidermal sheets
Source: J Cell Mol Med. 2020 Nov 8;24(23):14073–85. doi: 10.1111/jcmm.16019 (PMC7753989; doi:10.1111/jcmm.16019)

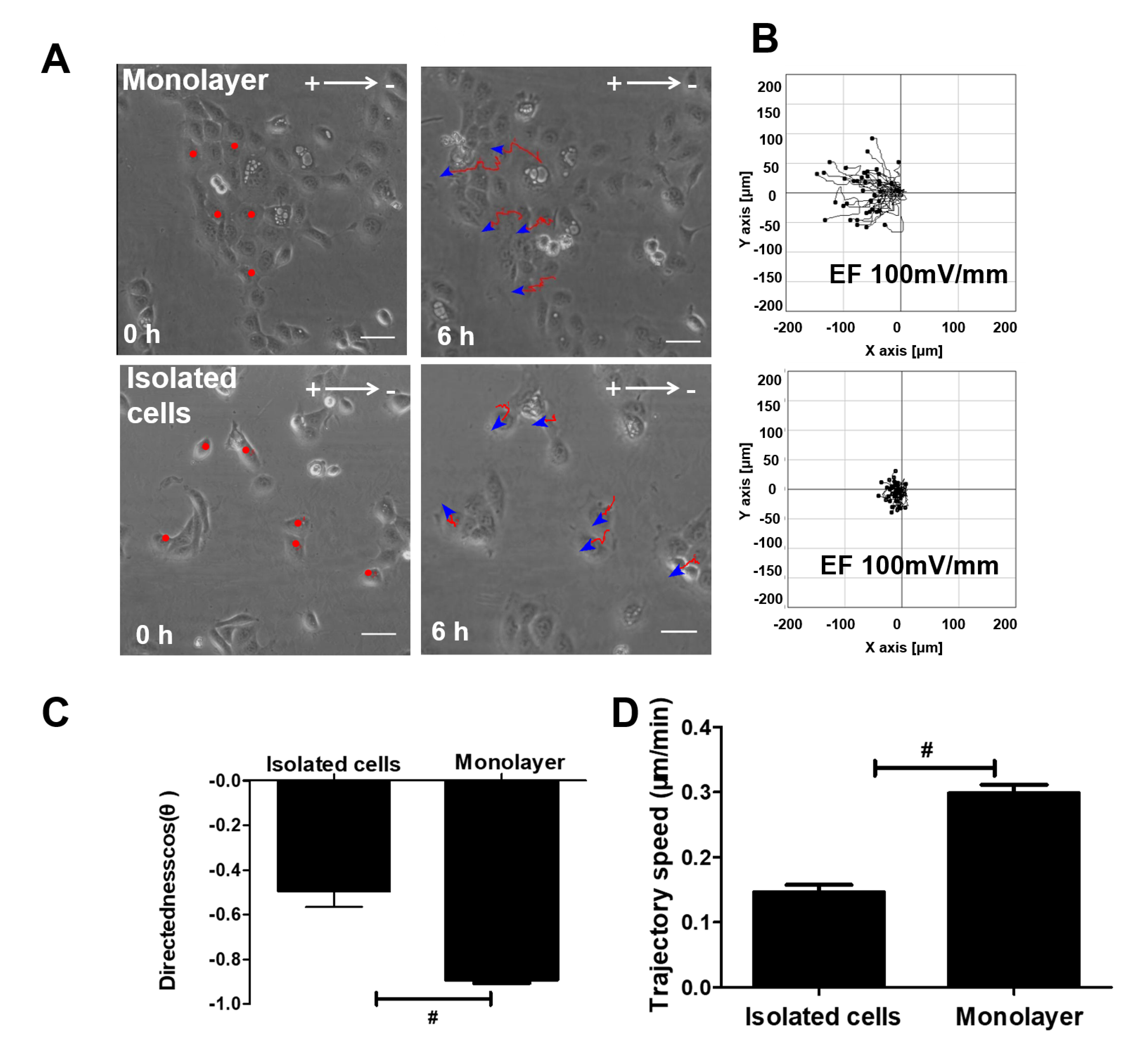

Supplement: Supplementary file 8 — Fig S1 [file JCMM-24-14073-s008.tif]

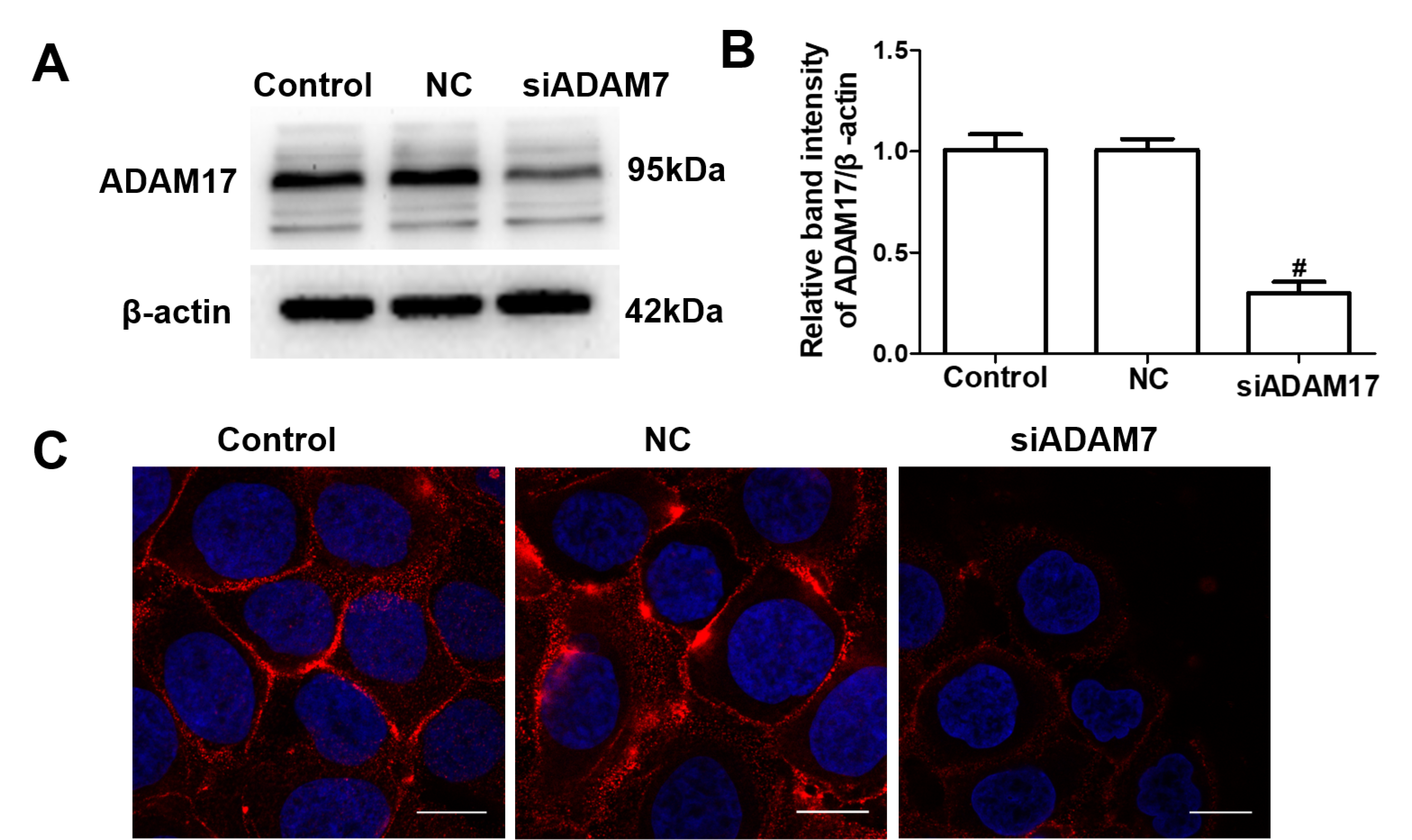

Supplement: Supplementary file 9 — Fig S2 [file JCMM-24-14073-s009.tif]

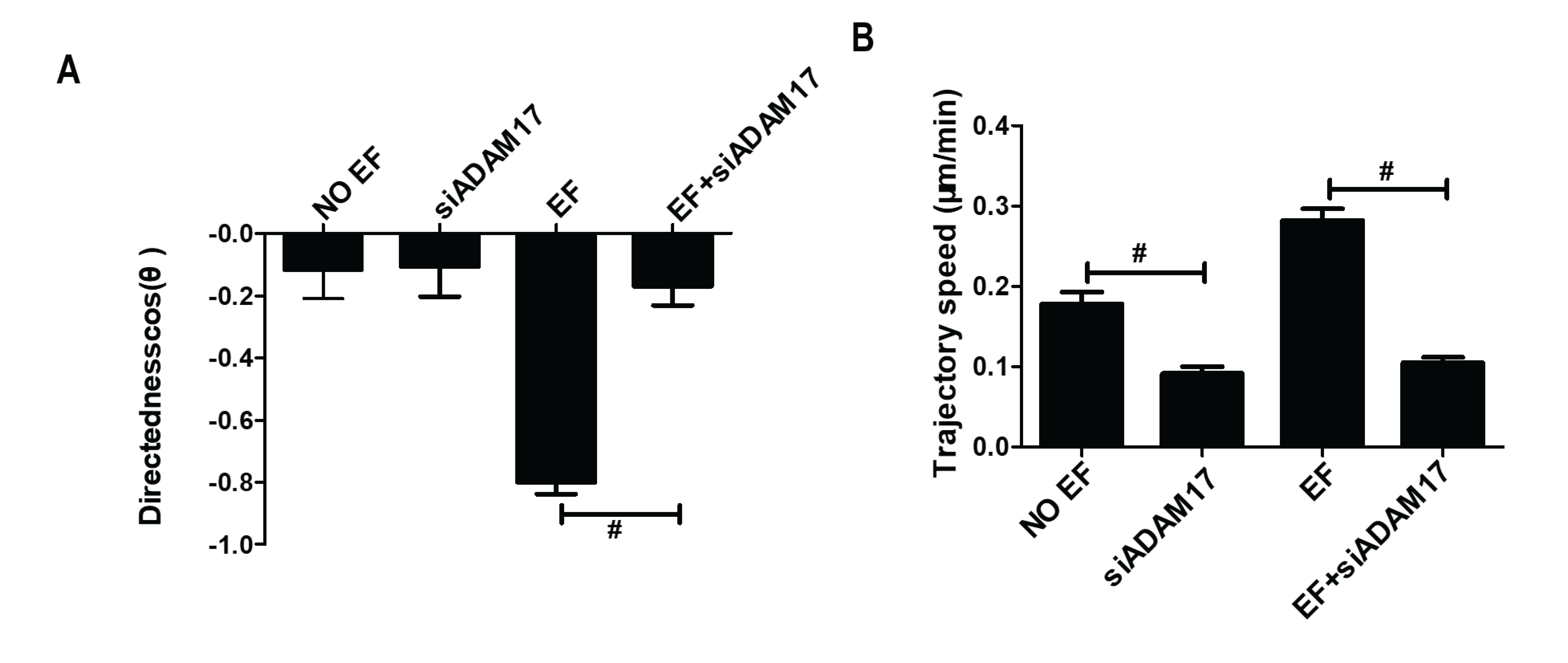

Supplement: Supplementary file 10 — Fig S3 [file JCMM-24-14073-s010.tif]
